# MRI assessment of changes in adipose tissue parameters after bariatric surgery

**DOI:** 10.1371/journal.pone.0206735

**Published:** 2018-11-02

**Authors:** Stefanie Lehmann, Nicolas Linder, Ulf Retschlag, Alexander Schaudinn, Roland Stange, Nikita Garnov, Arne Dietrich, Andreas Oberbach, Thomas Kahn, Harald Busse

**Affiliations:** 1 Integrated Research and Treatment Center (IFB) AdiposityDiseases, Leipzig University Medical Center, Leipzig, Germany; 2 Department of Diagnostics, Fraunhofer Institute for Cell Therapy and Immunology, Leipzig, Germany; 3 Department of Diagnostic and Interventional Radiology, Leipzig University Hospital, Leipzig, Germany; 4 Department of Visceral, Transplantation, Thoracic and Vascular Surgery, Section of Bariatric Surgery, Leipzig University Hospital, Leipzig, Germany; 5 Department of Cardiac Surgery, Ludwig-Maximilians-University, Munich, Germany; Medical University Innsbruck, AUSTRIA

## Abstract

Bariatric surgery and other therapeutic options for obese patients are often evaluated by the loss of weight, reduction of comorbidities or improved quality of life. However, little is currently known about potential therapy-related changes in the adipose tissue of obese patients. The aim of this study was therefore to quantify fat fraction (FF) and T1 relaxation time by magnetic resonance imaging (MRI) after Roux-en-Y gastric bypass surgery and compare the resulting values with the preoperative ones. Corresponding MRI data were available from 23 patients (16 females and 7 males) that had undergone MRI before (M0) and one month after (M1) bariatric surgery. Patients were 22–59 years old (mean age 44.3 years) and their BMI ranged from 35.7–54.6 kg/m^2^ (mean BMI 44.6 kg/m^2^) at M0. Total visceral AT volumes (V_VAT-T_, in L) were measured by semi-automatic segmentation of axial MRI images acquired between diaphragm and femoral heads. MRI FF and T1 relaxation times were measured in well-defined regions of visceral (VAT) and subcutaneous (SAT) adipose tissue using two custom-made analysis tools. Average BMI values were 45.4 kg/m^2^ at time point M0 and 42.4 kg/m^2^ at M1. Corresponding V_VAT-T_ values were 5.94 L and 5.33 L. Intraindividual differences in both BMI and V_VAT-T_ were highly significant (p<0.001). Average relaxation times T1_VAT_ were 303.7 ms at M0 and 316.9 ms at M1 (p<0.001). Corresponding T1_SAT_ times were 283.2 ms and 280.7 ms (p = 0.137). Similarly, FF_VAT_ differences (M0: 85.7%, M1: 83.4%) were significant (p <0.01) whereas FF_SAT_ differences (M0: 86.1, M1: 85.9%) were not significant (p = 0.517). In conclusion, bariatric surgery is apparently not only related to a significant reduction in common parameters of adipose tissue distribution, here BMI and total visceral fat volume, but also significant changes in T1 relaxation time and fat fraction of visceral adipose tissue. Such quantitative MRI measures may potentially serve as independent biomarkers for longitudinal and cross-sectional measurements in obese patients.

## Introduction

In western countries, obesity is a growing healthcare problem and also associated with metabolic and cardiovascular diseases. Adipose tissue (AT) is a metabolically active organ that is highly affected by obesity and chronic low-grade inflammatory processes in obese AT have been reported to contribute significantly to the development of obesity-related morbidities [1]. One focus of medical research is therefore the development of effective anti-obesity therapies. Bariatric surgery and Roux-en-Y gastric bypass (RYGB) surgery in particular have shown significant therapeutic effects in the treatment of morbid obesity [2]. Besides the marked weight loss and reduced amounts of abdominal visceral (VAT) [3–5] and subcutaneous (SAT) [3–5] adipose tissue, bariatric surgery may reduce co-morbidities like type 2 diabetes and cardiovascular diseases [6,7]. The underlying mechanisms that ultimately improve the health of patients after such interventions are still not well understood. Intraoperative and follow-up biopsies are a direct way to assess the inflammatory [8,9] or morphological [10] response of adipose tissue to excessive weight. On the downside, invasive tissue sampling requires good patient compliance and has side effects, in particular for biopsies in visceral fat regions. There is consequently a need for a relatively simple, safe and reliable technique to assess and monitor the effects of bariatric surgery on adipose tissue.

Over the last decade, different magnetic-resonance techniques have been applied to characterize adipose tissue in vivo. Magnetic resonance spectroscopy (MRS) essentially probes the distribution of fatty acid components [11–13] whereas chemical shift-encoded water-fat MR imaging (MRI) may quantify the fat fraction (FF) in white or brown adipose tissue [14–16]. In a recent study, measurements of T1 relaxation times in adipose tissue have revealed significant differences between obese and healthy lean subjects [17].

The purpose of this work was to assess T1 relaxation time and fat fraction changes by MRI in visceral and subcutaneous AT of patients before and after Roux-en-Y gastric bypass (RYGB) surgery.

## Materials and methods

This study was performed within the Integrated Research and Treatment Center on adiposity diseases (IFB *AdiposityDiseases* at Leipzig University Hospital, Leipzig, Germany) and approved by the institutional review board (IRB No. 363-11-ff-07032011, Faculty of Medicine, University of Leipzig). Written informed consent was obtained from all subjects.

All patients underwent standard laparoscopic RYGB with a small pouch (20 cm^3^), an alimentary limb of 150 cm and a biliopancreatic limb of 50 cm. Surgery was performed following applicable German and IFSO guidelines. Twenty-three patients (16 females and 7 males, mean age 44.3 years, mean BMI 45.4 kg/m^2^, both at M0) were included. Anthropometrics were recorded for both MRI time points.

All patients were examined in a 1.5-T MRI (Philips Achieva XR, Best, Netherlands) at time points before (M0) and one month (M1) after surgery. Fat volumetry involved axial in-phase/opposed-phase gradient echo images with 10 mm thickness and 0.5 mm gap that were acquired between diaphragm and femoral heads (typically 50 slices) in breath-hold technique (expiration). Other imaging parameters were: repetition time (TR) 76 ms, first/second echo time (TE_1_/TE_2_) 2.3/4.6 ms, flip angle (FA) 70, slice thickness (ST) 10 mm, interslice gap (ISG) 0.5 mm, field of view (FOV) 530 mm × 530 mm, acquisition matrix (M_ACQ_) 216 × 177, reconstruction matrix (M_REC_) 480 × 480. The total acquisition time (TA) was 10 x 16 seconds plus the intervals for breathing. All MRI data were acquired with the integrated whole-body coil.

Total abdominal VAT volumes (V_VAT_) were then evaluated by a semiautomatic fat segmentation tool (under Matlab). Automatic contouring used k-means clustering to detect the outer body (SAT) boundary and an iterative active-contours (snakes) algorithm in combination with region growing to define an inner SAT as well as a VAT boundary. A detailed description can be found in a previous work [18]. Incorrect portions of the automatic contours were simply corrected for by redrawing along the proper tissue boundary–effectively overwriting the wrong portions. Quantification of fat volumes involved a histogram-based analysis of the area under the fat-related peak structures (at higher signal intensities). This approach involves slightly more user interaction but also eliminates the need for highly accurate VAT boundary definition. Inter-observer reliability and reproducibility of that method have previously been found to be high [19]. Total SAT volumes were not considered because the SAT regions of several patients extended beyond the scanner's field of view.

**Fig 1:**
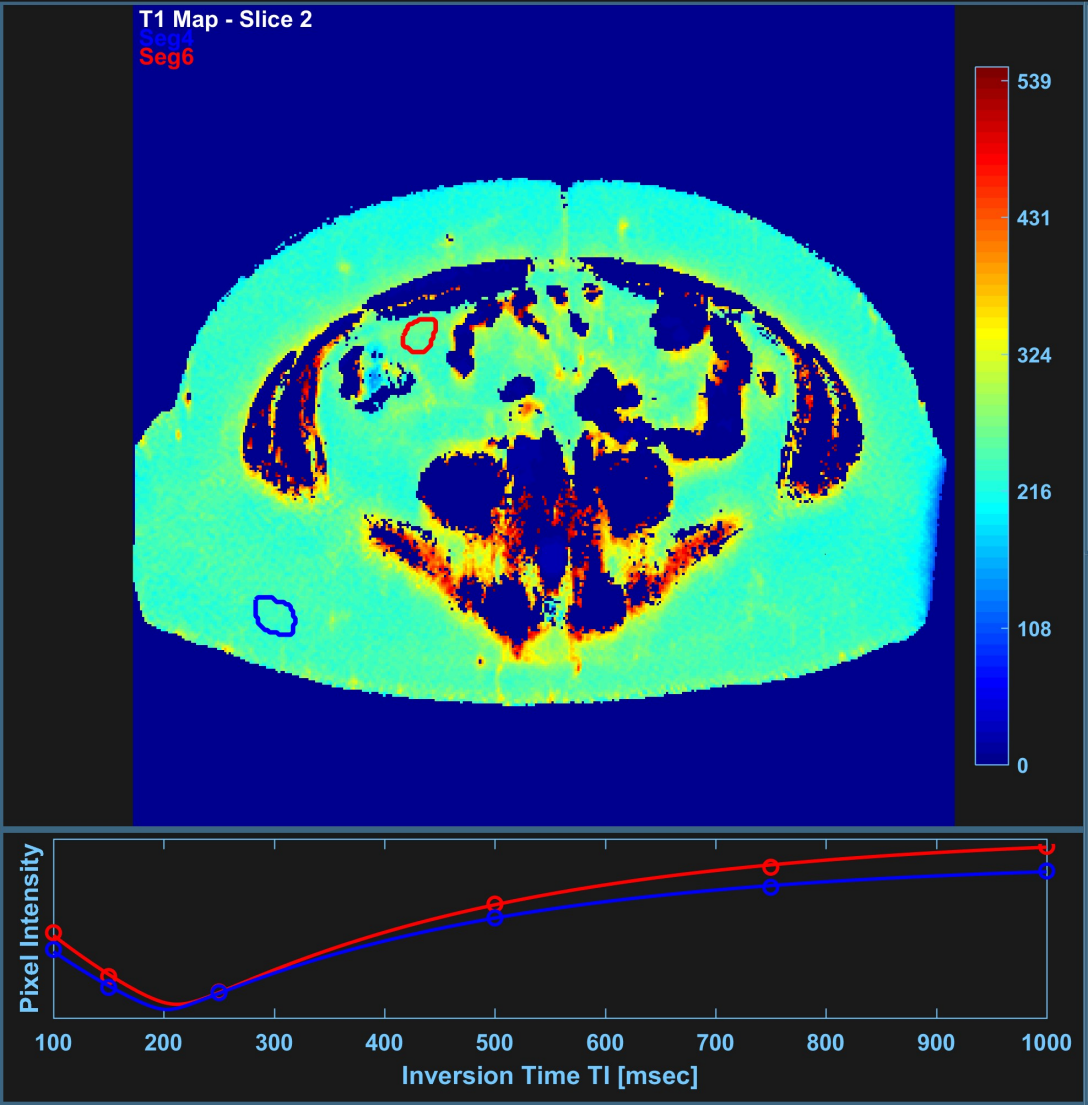
Screenshot of custom-made software tool for the quantification of T1 relaxation times in selected regions of interest. Display shows presurgical axial T1 map of an obese, 31-year-old female patient with a BMI of 41.1 kg/m^2^. T1 values were determined in VAT (174 pixels) and SAT ROI (237 pixels) by fitting mean signal intensities (SI) in arbitrary units (a.u.) of single-shot fast spin-echo sequence with inversion recovery preparation at different inversion times TI to model function [17].

T1 relaxation times were derived from a single-shot fast spin-echo sequence (TR/TE = 5,000/60 ms, FOV 500 mm × 500 mm, M_ACQ_ 296 × 295, ST 10 mm) with inversion recovery preparation at different inversion times TI (100, 150, 250, 500, 750 and 1,000 ms) [20]. A custom-made software tool under Matlab tool was used to compute the T1 time by fitting the TI-dependent signal intensities *SI* to the following model function

$$SI(TI)=\sqrt{S_{0}^{2}\;(1-2∙e^{-(\frac{TI}{T1})}+e^{-{\left(\frac{T_{SAT}}{T1}\right)}^{2}}+C^{2})}$$ (1)

with calibration constant *S*_0_, noise-related constant *C* and saturation time T_SAT_ (4,500 ms). Further technical details can be found in the original work by de Bazelaire et al. [21]. Fig 1 shows an example of a resulting T1 map with SAT and VAT regions of interest (ROI) used for quantification. VAT ROIs were generally placed within the mesentery at some distance away from signal heterogenities, sparing intestines, peritoneum and vessels.

**Fig 2:**
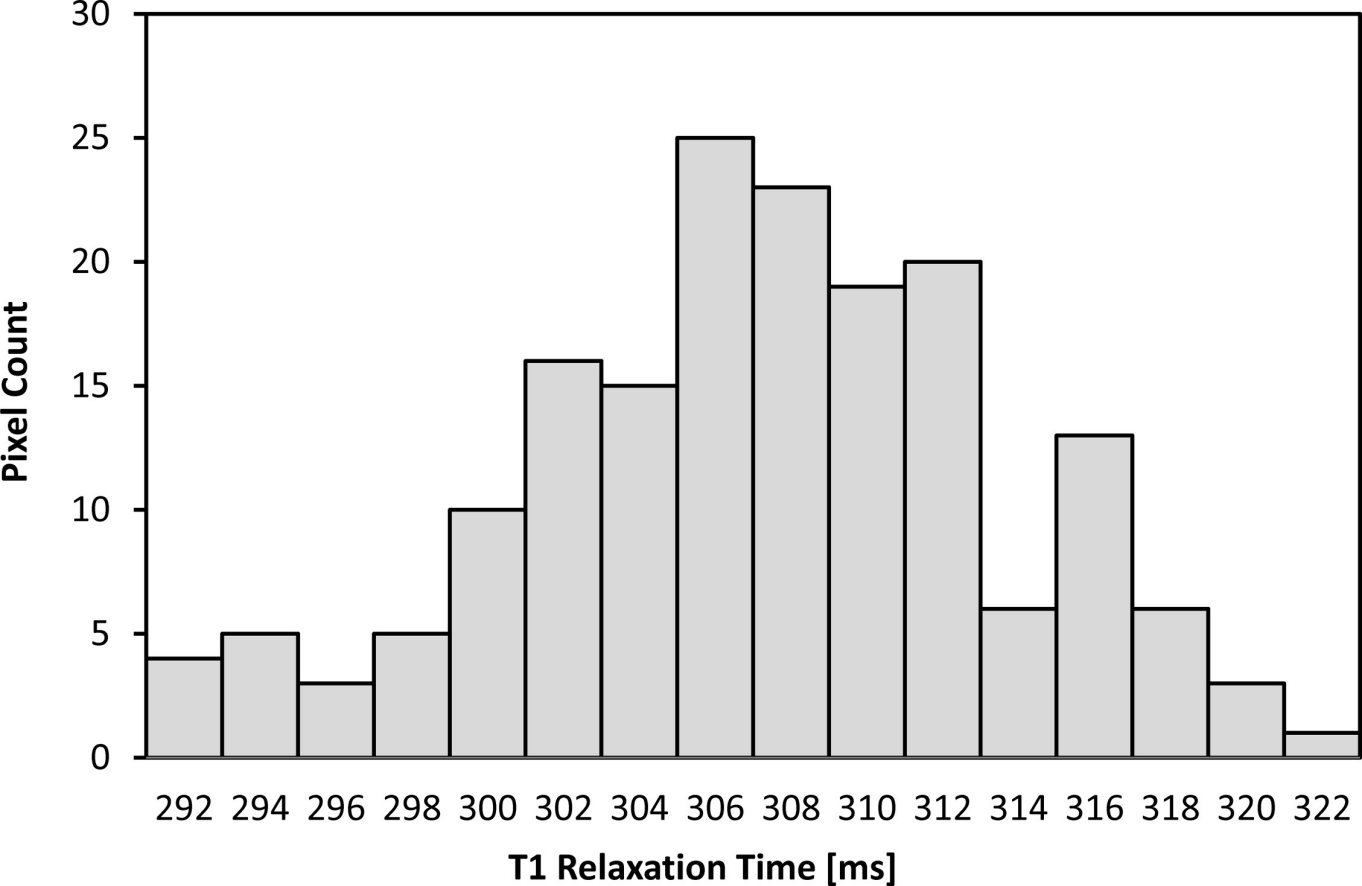
Distribution of individual T1 times for VAT ROI from Fig 1. Corresponding coefficient of variation CV_T1_ is taken as a quantitative estimate for the reliability of ROI-based T1 measurement, here 2.1%.

Reliability of ROI-based T1 measurement was then estimated by inspecting the distribution of individual T1 times and computing the coefficient of variation CV_T1_ as the ratio of mean TI intensity μ_T1_ and standard deviation σ_T1_ (Fig 2). Reported T1 times for VAT and SAT are then mean values μ_T1_ of the respective ROI.

For evaluation of the so-called proton density fat fraction in adipose tissue, chemical shift-encoded water-fat MRI was carried out using a multiecho Dixon sequence (3D spoiled gradient echo, TR 8.7 ms, TE 0.92–7.55 ms, 11 echoes, echo spacing 0.66 ms, FOV 384 mm × 288 mm, M_ACQ_ 128 × 96, FA 5°, 18 slices, ST 10 mm, TA 19.3 s) in breathhold technique. Fat fraction (FF) and T2* time were then estimated by least-squares fitting the mean ROI signal intensities at all echo times TE to the model function

$$SI(TE)=|S_{W}+e^{i∙2\pi \Delta f∙TE}∙S_{F}|∙e^{-TE/T2*}$$ (2)

with *S*_W_ and *S*_F_ as complex signal contributions of water and fat, respectively, and Δ*f* as the frequency difference between the main lipid (1.3 ppm) and water peaks (4.7 ppm), approximately 220 Hz at 1.5 T. Computations were performed under Matlab (MathWorks, Natick, MA) and verified against an independent code under IDL (Exelis Visual, Boulder, CO).

Statistical evaluation was performed with SPSS (Version 24, IBM SPSS). Statistical significance was assumed at p<0.05. All data sets–six individual parameters at both time points (M0 and M1) as well as six percent changes (M1 vs. M0)–were tested for normal distribution using Shapiro-Wilk tests. Percent changes relative to M0 were then analyzed with a one sample Wilcoxon signed-rank test against a hypothetical median of 0% or with a one-sample t-test against a 0% mean depending on the p-values of the Shapiro-Wilk tests.

## Results

**Fig 3:**
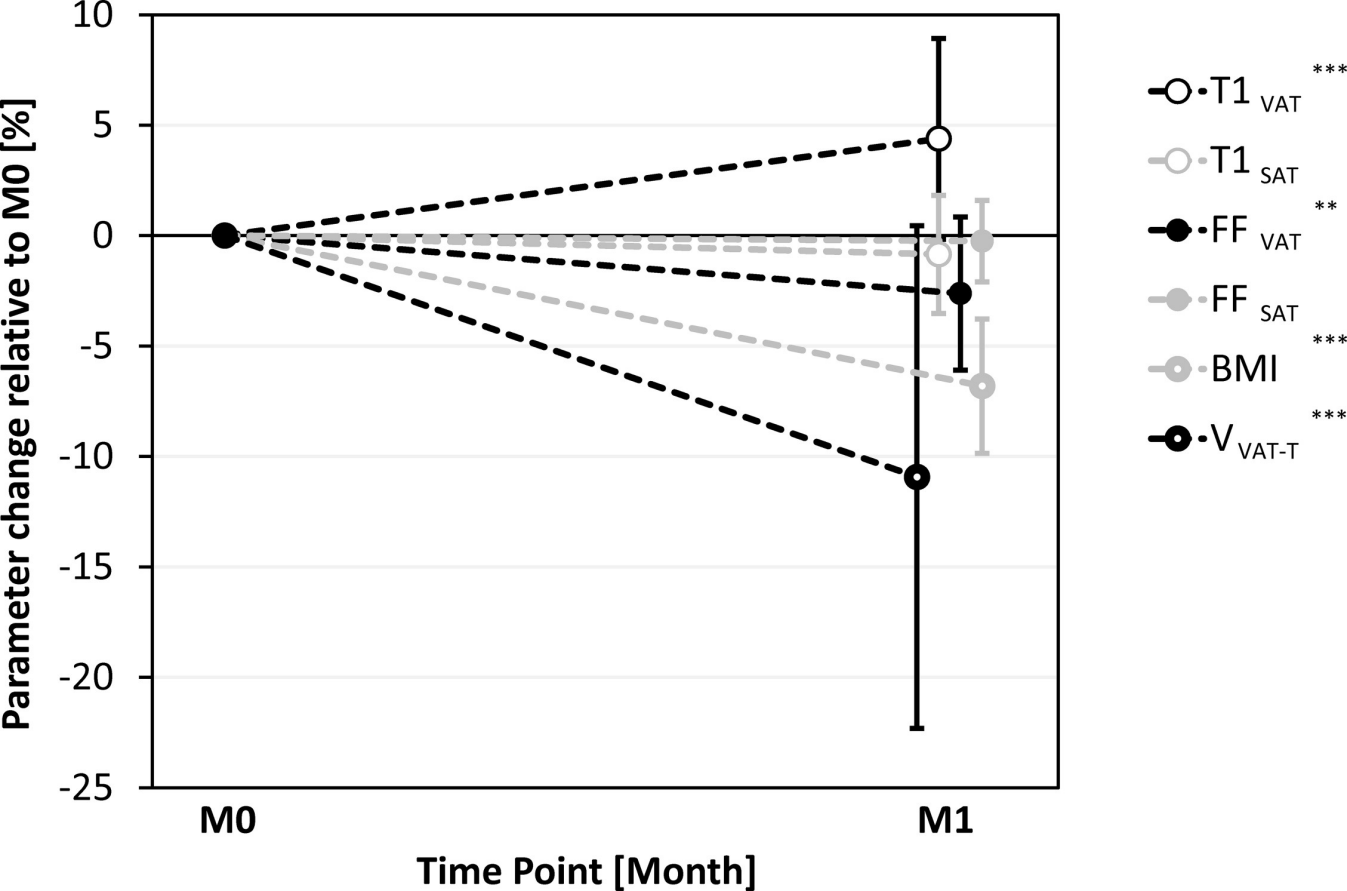
Plot of relative changes in adipose tissue parameters for 23 obese patients one month after bariatric surgery. Symbols show mean percent changes in BMI, total abdominal VAT volume (V_VAT-T_), regional fat fraction in visceral and subcutaneous adipose tissue (FF_VAT_ and FF_SAT_), and corresponding T1 relaxation times (T1_VAT_ and T1_SAT_) one month after (M1) relative to before bariatric surgery (M0). Error bars indicate 95% confidence intervals. Asterisks denote level of significance: * (p<0.05), ** (p<0.01), *** (p<0.001). Some symbols have been slightly offset to improve readability.

Table 1 provides a summary of the main results. All parameter sets but percent V_VAT-T_ change were in line with the assumption of a normal distribution. Fig 3 shows a plot of the percent changes for all six parameters after one month (M1 vs. M0). The BMI reduction of nearly –7% was significant (p<0.001). The most prominent relative effect of nearly –11% was observed for total VAT volume, again significant (p<0.001). T1 coefficients of variation (CV) over all subjects and both time points were 1.8±0.3% (range 1.1–2.4%) for SAT and 2.2±0.8% (range 1.3–4.8%) for VAT.

**Table 1:** Body mass index (BMI) and MRI-derived parameters of obese patients before (M0) and one month after bariatric surgery (M1).

Measure	Month 0 [M0]	Month 1 [M1]
**BMI [kg/m**^**2**^**]**	45.4 ± 5.7	42.4 ± 5.9 (–6.8 %) ***
**V**_**VAT-T**_ **[L]**	5.94 ± 2.24	5.33 ± 2.21 (–10.9 %) ***
**T1**_**VAT**_ **[ms]**	303.7 ± 9.7	316.9 ± 14.1 (+4.4 %) ***
**T1**_**SAT**_ **[ms]**	283.2 ± 8.2	280.7 ± 10.4 (–0.9 %)
**FF**_**VAT**_ **[%]**	85.7 ± 2.8	83.4 ± 2.9 (–2.6 %) **
**FF**_**SAT**_ **[%]**	86.1 ± 1.8	85.9 ± 2.0 (–0.2 %)

V_VAT-T,_ total visceral VAT volume; FF, fat fraction; T1, T1 relaxation time

p≥0.05

*, p<0.05

**, p<0.01 and

***, p<0.001.

**Fig 4:**
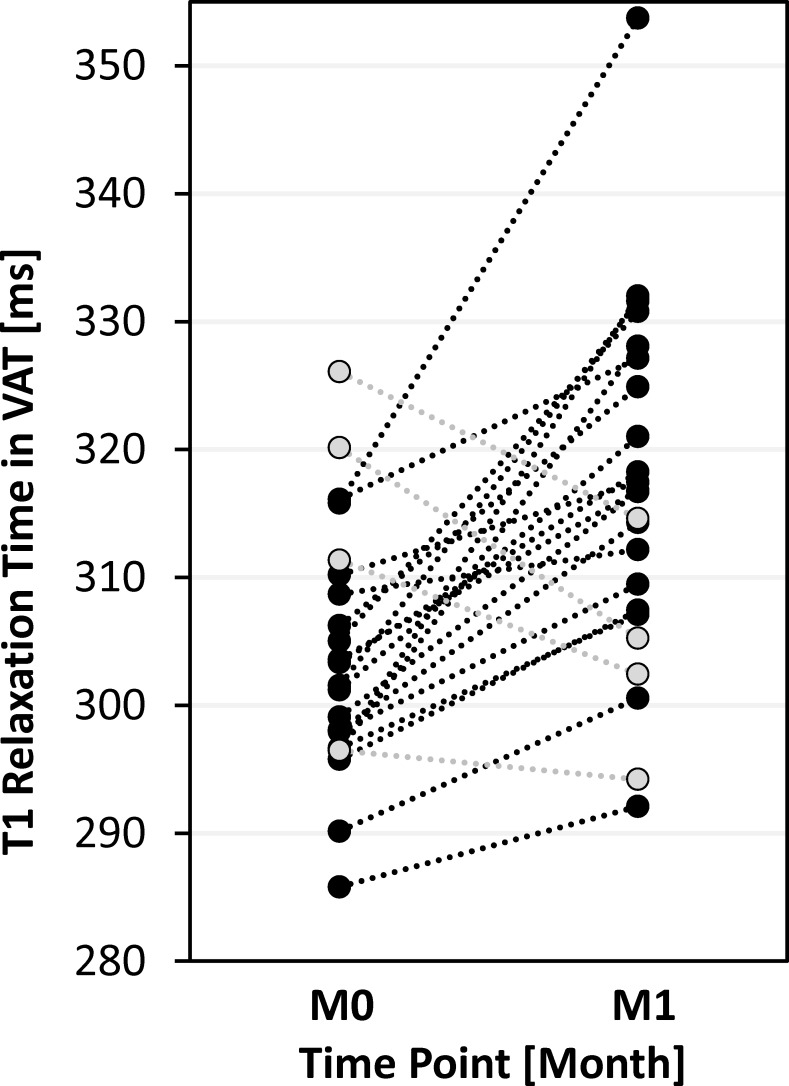
Intra-individual T1 relaxation times (at 1.5 T) in visceral adipose tissue (VAT) of 23 obese patients one month after (M1) relative to before bariatric surgery (M0). Black circles indicate patients with increased T1 time (n_+_ = 18) and gray circles those with either reduced (n_–_ = 4) or same T1 time (n_0_ = 1). Overall increase was highly significant (p<0.01).

At the ROI level, T1 relaxation times were significantly longer in VAT (+4.4%, p<0.001) but not in SAT (–0.9%, p = 0.141). Fig 4 illustrates the intraindividual T1 changes in VAT. The early relative changes in fat fraction were significant for VAT (–2.6%, p<0.01) but not for SAT (–0.2%, p = 0.564). Detailed information is provided in S1 Table.

## Discussion

Quantification of VAT and SAT by MRI is increasingly used to monitor body fat parameters during obesity interventions [4,5]. Previous studies have found changes in both VAT volume and body composition using anthropometry [22], dual X-ray absorptiometry [23,24], computed tomography [23] or MRI [25].

The presented in-vivo MRI work is among the first to report on significant early changes in VAT parameters one month after RYGB, specifically in regional fat fraction (decrease) and T1 relaxation time (increase). So far, VAT composition has been studied mainly ex vivo, either by biopsy or autopsy. Tissue samples of adipose tissue during elective laparoscopy have indicated that specific metabolic genetic programming in VAT and SAT was associated with BMI. That data suggested that BMI promotes de novo lipogenesis in VAT [26].

In contrast, our data revealed no significant differences in SAT parameters. It is still largely unknown which structural and functional processes in adipose tissue are responsible for the observed changes in FF and T1 after surgery also because of insufficient data from dedicated biopsy studies. Bariatric surgery has been reported to improve the inflammatory state [8,9] and reduce adipocyte volume [10]. Another study has reported a significant correlation between T1 time and mean adipocyte cell size in SAT [17]. It is also worth noting that unlike tissue samples, MRI information can be obtained non-invasively but interpretation is generally complicated by the difference in scale–microscopic versus macroscopic.

Water-fat MRI studies in human subjects have generally focussed on brown adipose tissue but some have also addressed white adipose tissue [14–16]. Leporq et al. [16], for example, have seen differences in FF_VAT_ between two healthy obese (84.3%) and three non-obese (BMI < 30 kg/m^2^) subjects (89.5%) with liver steatosis while the corresponding FF_SAT_ values were the same (93.0%). In a different study, Hu et al [14] have observed a higher mean FF_SAT_ for overweight or obese children (90.3%, n = 22) than for children with normal weight (80.9%, n = 17). An earlier comparison of relaxation times in adipose tissue had found T1 times in healthy lean subjects (mean BMI of 21.5 kg/m^2^, n = 10) to be larger than in severely obese patients (41.4 kg/m^2^, n = 20) for both VAT (360 versus 294 ms, +66 ms) and SAT (301 versus 275 ms, +26 ms) [17].

The individual changes in BMI, V_VAT_, FF_VAT_ and T1_VAT_ found here were all significant. The largest relative effect was seen for V_VAT_ (about -11%) followed by BMI (-7%). In addition, the mean absolute values (304 and 317 ms for VAT, 283 and 281 ms for SAT) happen to fall within the previously measured range between healthy lean subjects and severely obese patients (294–360 ms for VAT and 275–301 ms for SAT). The T1_VAT_ increase (+4.4% or +13.2 ms) is therefore in line with a shift towards T1 values previously measured in healthy lean subjects. In contrast, the measured T1_SAT_ and FF_SAT_ differences were not significant, which is most likely explained by the more subtle SAT effect. Also note that the intraindividual BMI difference was just 3 kg/m^2^ after one month compared to nearly 20 kg/m^2^ between the subject groups referred to above.

This retrospective study is limited by various factors. The relatively small number of patients from a single institution precluded subgroup analyses for variables like age or gender. Furthermore, the analysis did not involve histological validation of our results because repeated biopsies during follow-up examinations are limited for ethical and medical reasons. Another limitation is given by the consideration of the main fat peak only (at 1.3 ppm) when modeling the TE dependence of the MR signal intensity (Eq 2). A previous phantom study has shown MRI estimation of FF [27] to be accurate but has assumed eight individual fat resonances instead. Wang et al. have studied the effect of the number of included peaks in more detail [28] and have found that the use of one peak leads to an underestimation of the obtained fat fraction. In a single-peak model, a change in FF may theoretically also be due to a mere change in fat composition and could then be misinterpreted. Further studies on a larger number of subjects or with the addition of suitable histopathologic markers, such as adipocyte size or inflammation markers, are required to improve statistical power and understanding of the underlying processes.

In conclusion, there were detectable MRI changes in T1 relaxation time and fat fraction of VAT one month after bariatric surgery. Besides the widely used morphological measures derived from fat volumes, these potential MRI markers for the tissue response in obese patients deserve further attention.

## Supporting information

S1 Table The individual data for both timepoints M0 and M1. (XLSX)
